# Accuracy and efficiency define Bxb1 integrase as the best of fifteen candidate serine recombinases for the integration of DNA into the human genome

**DOI:** 10.1186/1472-6750-13-87

**Published:** 2013-10-20

**Authors:** Zhengyao Xu, Louise Thomas, Ben Davies, Ronald Chalmers, Maggie Smith, William Brown

**Affiliations:** 1 Queens Medical Centre, School of Life Sciences, Nottingham University, Nottingham NG7 2UH, UK; 2 School of Medical Sciences, College of Life Sciences and Medicine, University of Aberdeen, Institute of Medical Sciences, Aberdeen AB25 2ZD, UK; 3 Wellcome Trust Centre for Human Genetics, University of Oxford, Roosevelt Drive, Oxford OX3 7BN, UK; 4 Department of Biology, University of York, Wentworth Way, York YO10 5DD, UK

**Keywords:** Serine recombinases, Genome manipulation, DNA damage

## Abstract

**Background:**

Phage-encoded serine integrases, such as φC31 integrase, are widely used for genome engineering. Fifteen such integrases have been described but their utility for genome engineering has not been compared in uniform assays.

**Results:**

We have compared fifteen serine integrases for their utility for DNA manipulations in mammalian cells after first demonstrating that all were functional in *E. coli*. Chromosomal recombination reporters were used to show that seven integrases were active on chromosomally integrated DNA in human fibroblasts and mouse embryonic stem cells. Five of the remaining eight enzymes were active on extra-chromosomal substrates thereby demonstrating that the ability to mediate extra-chromosomal recombination is no guide to ability to mediate site-specific recombination on integrated DNA. All the integrases that were active on integrated DNA also promoted DNA integration reactions that were not mediated through conservative site-specific recombination or damaged the recombination sites but the extent of these aberrant reactions varied over at least an order of magnitude. Bxb1 integrase yielded approximately two-fold more recombinants and displayed about two fold less damage to the recombination sites than the next best recombinase; φC31 integrase.

**Conclusions:**

We conclude that the Bxb1 and φC31 integrases are the reagents of choice for genome engineering in vertebrate cells and that DNA damage repair is a major limitation upon the utility of this class of site-specific recombinase.

## Background

Serine integrases are phage-encoded site-specific recombinases that promote conservative recombination reactions between short (40-60 bp) DNA substrates located on the phage (phage attachment site, *attP*) and bacterial (bacterial attachment site, *attB*) chromosomes [1]. The product of *attP* × *attB* recombination is an integrated prophage flanked by two new recombination sites, *attL* and *attR*, each containing half sites derived from *attP* and *attB*. In the absence of accessory factors the integrases mediate unidirectional recombination between *attP* and *attB* with greater than 80% efficiency. In the presence of a phage-encoded accessory protein, the recombination directionality factor (RDF) the *attP* × *attB* recombination is inhibited and the *attL* × *attR* recombination is stimulated [2,3]. In this way integration (*attP* × *attB*) and excision (*attL* × *attR*) of the phage genome are under strict controls and in tune with the phage life cycles. The unidirectional activity, short substrate sites and functional autonomy of these recombinases has made them a useful complement to the widely used reversible recombinases of the tyrosine recombinase family such as Cre and Flp for genome engineering reviewed in [1]. In particular the unidirectional activity has made them valuable for the promotion of DNA integration by recombinase-mediated cassette exchange reactions and for the development of iterative recombination approaches [4-7]. To date five serine integrases derived from phages φC31 [8], φBT1 [9], Bxb1 [10,11] and R4 [11,12] have been shown to be capable of promoting site-specific integration of DNA into mammalian genomes while TP901-1 [13], A118, FC1 and φRV [14] have been shown to promote site-specific recombination in an extra-chromosomal environment in mammalian cells. With one exception these studies have, however, been carried out largely independently of one another, in different cell lines, cells of different species and using different protocols. The exceptional study was that of Yamaguchi and colleagues [11] who compared the activities of the φC31, Bxb1, TP901-1 and R4 integrases in mediating site-specific recombination into a human artificial chromosome (HAC) isolated in hamster cells. This study exploited a promoter trap strategy and thus relied upon selection to assay recombination products. Importantly, however, the products were not analyzed at the level of DNA sequence. It was therefore neither possible to determine the total level of recombination promoted by these different enzymes nor to determine the fraction of recombination events that had proceeded by reciprocal and conservative site-specific recombination.

The discovery that site-specific recombination mediated by the φC31 integrase is sometimes accompanied by DNA damage in vertebrate cells identified [15] posed the question as how far integrase associated DNA damage limits the use of the serine integrases as genome engineering reagents. Damage of the type seen with the φC31 integrase, has not been detected with the tyrosine recombinases, is likely to be a consequence of the DNA cleavage and strand exchange mechanism of the serine recombinases (reviewed in [16]). The recombination pathway begins with binding of integrase to the attachment sites, which are then brought together by protein:protein interactions to form a synaptic tetramer. The reaction then proceeds by the formation of concerted double strand breaks in both of the DNA substrates prior to subunit rotation and recombination. It seems likely that the damage that accompanies the activity of these recombinases in vertebrate cells arises as a consequence of these double strand breaks being recognized by the mammalian cell double strand break repair pathways. It should be noted that to date no damage has ever been observed to accompany the action of the serine integrases in bacteria and it may therefore be the eukaryotic chromatin environment or the nature of the mammalian repair pathways that leads to the damage seen in mammalian cells. The frequency and extent of the damage would seem likely to reflect the time spent by the integrases in the covalently linked, cleaved DNA complex as this is most likely to be the target for the repair pathways.

In total there are fifteen phage-encoded serine integrases for which both of their attachment sites are known. Nine of these fifteen integrases have been characterised in reactions in mammalian cells, *E. coli* or *in vitro* (φC31 [17], Bxb1 [18], φBT1 [19], φC1 [14,20], MR11 [21], TP901-1 [22], R4 [12], A118 [14], and φRV [14], [23]) while six (TG1, φ370.1 [24], Wβ [25], BL3, SPBc and K38) have not yet been shown to be active outside their native hosts. In total there are ten integrases whose utility as tools for integrating DNA into mammalian genomes has not been investigated. We have therefore set out to rank the activities of all fifteen of these serine integrases for which the sites are known by the criteria of both accuracy and efficiency in two different mammalian cell lines; human HT1080 cells and mouse ES cells. These studies have provided us with a clear rank order for the utility of this important class of enzymes as tools for vertebrate genome engineering with Bxb1 integrase mediating the most efficient and accurate site-specific recombination in this heterologous environment. The φC31 integrase comes a close second. For the remaining integrases, we demonstrate that DNA damage is an important factor in limiting the utility of members of this class of enzymes for promoting integration reactions in mammalian cells.

## Results and discussion

### Fifteen unidirectional 'phage integrases are active in *E. coli*

**Figure 1:**
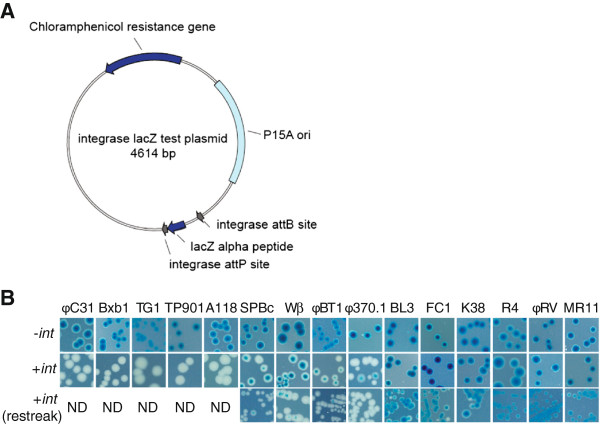
**Assaying integrase activity in *E.coli. *A**. The reporter plasmid used to assay activity of the integrases in *E. coli*. This plasmid, derived from pACYC184, contains *lacZα* gene encoding the LacZα peptide flanked by integrase attachment sites. The intact reporter plasmid confers β-galactisidase activity on a strain containing the ΔlacZM15 allele and therefore the colonies appear blue on agar containing presence of X-Gal and IPTG. Active integrase promotes site-specific recombination between the *attP* and *attB* sites resulting in deletion of the *lacZα* gene and the colonies appear white. **B**. The appearance of *E.coli* containing the reporter plasmid with or without an integrase expression plasmid in the presence of X-Gal and IPTG.

Fifteen unidirectional phage integrases have been described for which the attachment sites are known. We first set out to confirm that each integrase and their cognate attachment sites were active by expression of each integrase gene in *E. coli* in the presence of a reporter plasmid. Fifteen reporter plasmids (conferring chloramphenicol resistance) were constructed in which the *lacZα* gene was flanked by *attB* and *attP* sites for each of the respective integrases (Figure 1A). These plasmids conferred exclusively blue colony when transformed into an *E. coli* strain containing the *lacZΔM15* mutation and plated on selective agar in the presence of X-gal and IPTG (Figure 1B, top row). We next introduced genes encoding each one of the integrases tagged at the N-terminal end by a StrepII tag and at the C-terminal end by a SV40 large T antigen nuclear localization signal into the *E. coli* expression plasmid pET21a (conferring ampicillin resistance). Initially we introduced the integrase expression plasmids into DH5α *E. coli* K12 strains containing a cognate reporter plasmid and scored the transformants as white or light blue, indicative of recombination, or blue, indicative of no recombination. Active integrases were from phages φC31, Bxb1, TG1, TP901-1, A118, SPBc, Wβ, φBT1 and φ370.1 in this assay (Figure 1B, middle row). The transformants containing integrase genes that only gave rise to blue or light blue colonies were picked and restreaked to single colonies and white colonies were observed from strains expressing BL3, FC1 and K38 integrases whereas no white segregants were observed from strains containing MR11, φRV and R4 *int* genes (Figure 1B, bottom row).

As the expression of the integrases might be limiting in bacteria containing the MR11, φRV and R4 integrase genes, we repeated the recombination assay in *E. coli* BL21(DE3), a strain that should over-express the integrase genes on induction with IPTG. Competent BL21(DE3) containing the different reporter plasmids were prepared and the integrase containing expression plasmids introduced by transformation with selection for ampicillin and chloramphenicol resistance. Expression of integrase was induced by addition of IPTG to logarithmically growing cells and cultures were further incubated overnight at 20°C. Plasmid DNA was extracted from 1 ml of each culture and used to transform plasmid free DH5α scoring for white and blue colonies. All of the plasmids extracted from the *E. coli* BL21(DE3) cells expressing the integrases gave exclusively white colonies with the exception of strains that had contained MR11 or φRV integrases, which yielded 50% and 25% unrecombined plasmid, respectively (Table 1). The control BL21(DE3) strains that contained the reporter plasmids and the empty expression plasmid (pET21a) remained stable with no loss of the *lacZα* gene. Accurate site-specific recombination for all of the integrases was confirmed by PCR and sequencing of the *attL* sites recovered from plasmids present in the white colonies. Cells from the same cultures were used to assay the level of expression of the StrepII-tagged integrases by western blots (Table 1). A StrepII-tagged integrase of the expected molecular weight was detected from all the BL21 (DE3) strains expressing an integrase except those containing the Wβ, Bxb1, BL3 or R4 integrase genes. The amount of protein present was determined by comparing the intensities of the bands from the western blotting with those from a series of standards (Table 1). While there was no simple relationship between the amounts of protein detected in the cultures and the level of recombination, this experiment demonstrated that all the integrases and their attachment sites were active in *E. coli*.

**Table 1:** **In vivo recombination activity after overexpression of integrases in** ***E. coli*** **BL21(DE3)**

**Integrase**	**μg tagged protein/ml**^**1**^	**% recombination**^**2**^
Bxb1	ND	100
Wβ	ND	100
BL3	ND	100
φR4	ND	100
A118	0.6	100
TG1	2.7	100
MR11	2.9	50
φ370	4.1	100
SPBc	9.0	100
TP901-1	32.3	100
φRV	55.9	75
FC1	74.7	100
K38	319.6	100
φBT1	350.4	100
φC31	758.2	100

^1^The amount of StrepII tagged integrase detected in a Western blot from 1 ml of culture.

^2^The % of plasmid extracted from the cultures that had undergone recombination.

### Assaying the unidirectional phage integrases in vertebrate cells

**Figure 2:**
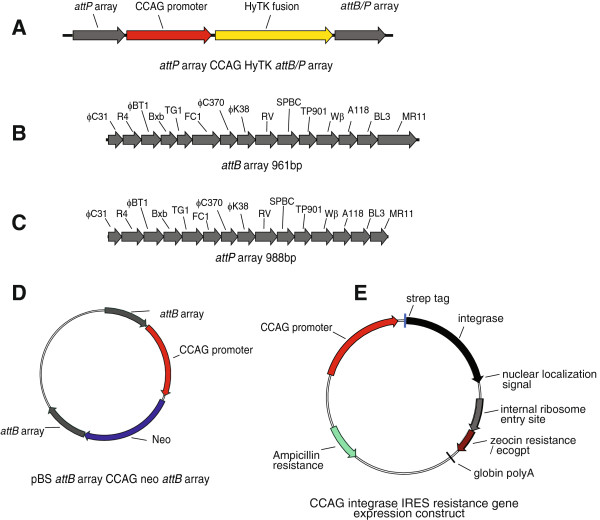
**Assay system for integrases in mammalian cells.** The reporter plasmid design **(A)** used to assay either site-specific deletion or integration promoted by serine integrases in vertebrate cells. In the deletion reporter construct, called *attP* array CCAG HyTK *attB* array, the counter selectable gene CCAG HyTK was placed between an array of *attB* sites **(B)** and an array of *attP* sites **(C)**. In the integration or recombinase-mediated cassette exchange constructs, the docking construct, *attP* array CCAG HyTK *attP* array, had the CCAG HyTK gene flanked by two arrays of *attP* sites and the reporter construct, termed *attB* array CCAG neo *attB* array **(D)** contained the CCAG Neo gene conferring resistance to G418 flanked by arrays of *attB* sites. The integrase expression constructs are shown schematically in **(E)** each containing an *int* gene modified at the 5′ and 3′ ends to encode a StrepII tag and a nuclear localization signal, respectively, and placed down-stream of a CCAG promoter and upstream of an internal ribosome entry site and a dominant selectable marker conferring either zeocin or xanthine resistance (ecogpt).

The activities of the integrases were then measured in vertebrate cells. In order to do this we constructed three reporter plasmids that could be used to assay the ability of all of the different integrases to mediate either deletion or integration in mammalian cells. Each of the reporter plasmids included arrays containing *attP* or *attB* sites for all 15 integrases arranged head-to-tail. For the deletion assay we used a plasmid, called '*attP* array CCAG HyTK *attB* array’ (Figure 2A), in which a counter selectable marker gene, a fusion between a gene conferring resistance to hygromycin and a herpes simplex virus thymidine kinase gene that confers sensitivity to the nucleoside analogue gancyclovir, HyTK, was placed between arrays of *attB* and *attP* sites in a head-to-tail orientation (Figures 2B and 2C). If integrase mediated recombination occurs between its cognate *attP* and *attB* sites flanking the HyTK gene, the cells become resistant to gancyclovir. The assay for integration activity was based upon the use of two plasmids. The first of these, called '*attP* array CCAG HyTK *attP* array’, contained the docking *attP* sites, flanking the counter selectable marker (Figure 2A) and the second, called '*attB* array CCAG neo *attB* array’, contained the incoming *attB* sites flanking a gene encoding resistance to the antibiotic G418 (Figure 2D). Cassette exchange between the incoming *attB* sites and the docking *attP* sites is expected to yield chromosomes containing *attR* and *attL* flanking the integrated *neo* gene and the cells display both gangcylovir and G418 resistance.

In both assays the integrases were expressed using plasmids (Figure 2E) in which each integrase gene had been codon optimized and tags placed at the 5′ and 3′ ends encoding the StrepII tag and the nuclear localization signal. An internal ribosome entry site (IRES) followed by an antibiotic resistance gene was placed downstream of each integrase gene.

**Figure 3:**
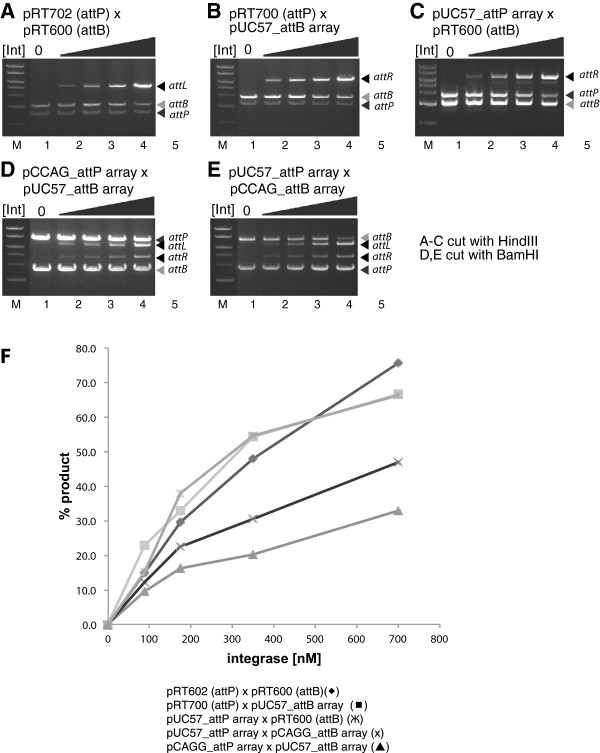
**Comparing the activity of** φ**C31 integrase on isolated attachment sites or attachment sites located in the multi-site arrays. A-E**. Assays φC31 integrase activity on attachment sites in different configurations. The indicated plasmids containing either *attB* or *attP* sites were incubated in the absence or in the presence of 0 nM (lane 1), 100 nM (lane 2), 200 nM (lane 3), 350 nM (lane 4) and 700 nM (lane 5) φC31 integrase. Recombination reactions were digested with either HindIII or BamHI and the products size fractionated by agarose gel electrophoresis. **F**. The data shown in A-E were analyzed densitometrically and the fraction of the substrate converted into product calculated as described in the materials and methods. This comparative analysis was carried out in two independent experiments.

The use of arrays of attachment sites in the reporter plasmids had three merits: Firstly it enabled a strategy that ensured that comparisons between the different integrases were not compromised by position effects arising as a result of the target sites for the different integrases being integrated at different positions in the vertebrate genome. Secondly it allowed us to determine whether there was any cross-reactivity between the different integrases and their attachment sites. Finally this approach proved to be efficient, requiring only a small number of cell lines to be used for all of the necessary assays. However as a precaution, we wanted to exclude the possibility that placing the attachment sites within an array altered their activity and so we compared the *in vitro* activity of one of the integrases, φC31 integrase, using substrates in which the attachment sites were either isolated or present in the arrays (Figure 3A-E). These experiments showed that the recombination efficiencies of the attachment sites were similar whether they were isolated or present in the arrays.

### Identification of eight unidirectional phage integrases promoting site-specific deletion in mammalian cells

Firstly we wanted to determine which of the fifteen integrases were active on substrates integrated into vertebrate genomes. The most sensitive way in which to detect activity is to assay recombination between *attB* and an *attP* sites in *cis* because the proximity of the sites favours the kinetics of synapsis, the process by which integrase bring the substrates together in a tetramer prior to DNA cleavage. We therefore transfected the construct designed to assay deletion, *attP* array CCAG HyTK *attB* array*,* into human HT1080 cells by electroporation and selected for stable transfectants using hygromycin. We screened 96 stably transfected clones for the integrity of the integrated DNA using PCR, checked for single copy integrants by restriction enzyme analysis and filter hybridization and then confirmed the integrity of the *attP* and *attB* arrays by sequencing. In this way we recovered four independent, stably transfected clones from two independent transfections, each containing a single copy of the integrated deletion reporter construct, *attP* array CCAG HyTK *attB* array.

In order to compare the activities of the different integrases we first transiently transfected 10^5^ HT1080 cells containing the *attP* array CCAG HyTK *attB* array reporter with expression plasmids for each of the integrases using Lipofectamine and assayed for recombination activity by selecting for resistance to gancyclovir, a drug which is selectively toxic for cells expressing the HyTK fusion. None of the integrases gave a significant increase in the number of gancyclovir-resistant cells as compared to the empty expression vector (Adiitional file 1: Table S3) and so we assayed pools of the resistant cells for the presence of the recombinant *attR* site using PCR. Recombination activity was detected in populations of cells transfected with the R4, φC31, φBT1, Bxb1, SPBC and Wβ integrase expression constructs (not shown). However the low level of activity overall made it impossible to conclude anything about the integrases that did not yield *attR* in the PCR reactions as the integrases may be active but causing damage that removed the *attR* primer binding sites, may simply be slow in promoting site-specific recombination or completely inactive. Moreover the variability of the relative numbers of gancyclovir resistant clones generated in different experiments made an accurate comparison between the active integrases impractical.

**Figure 4:**
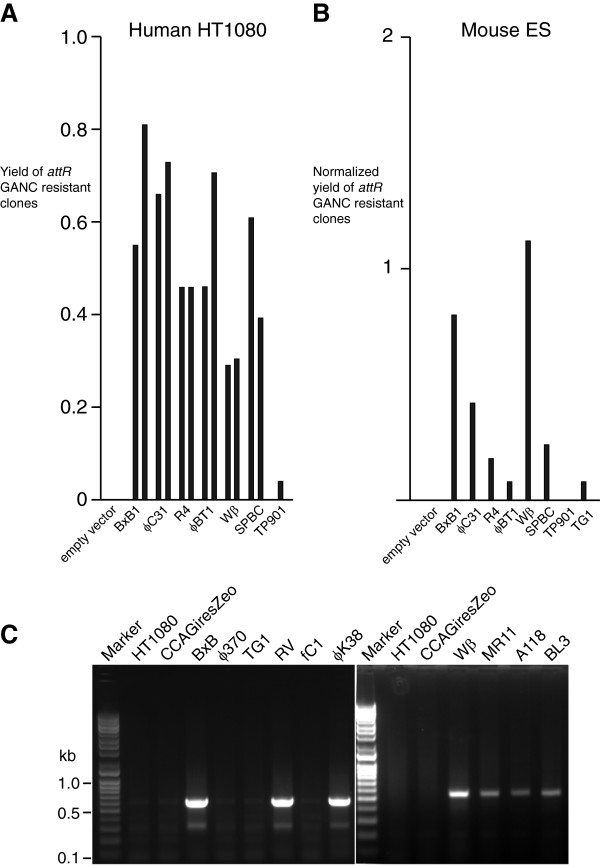
**Comparing the activity of fifteen serine recombinases in human HT1080 cells and in mouse ES cells. A**. HT1080 cells containing a single integrated *attP* array CCAG HyTK *attB* array reporter construct were stably transfected with an integrase expression plasmid selecting for antibiotic resistance. The transfected clones were then subjected to gancyclovir selection to identify those that had lost the CCAG HyTK marker gene and then screened for recombinant *attR* sites to identify those that had undergone integrase-mediated site-specific deletion of CCAG HyTK. The two bars shown for each integrase reflect the results of two independent experiments. (Additional file 1: Table S4). None of the other eight integrases yielded recombinant gancyclovir resistant clones and they are not shown in this part of the figure. **B**; ES cells containing a *attP* array CCAG HyTK *attB* array reporter integrated at the ROSA26 locus were transiently co-transfected with an integrase expression plasmid and a linearized PGKneo construct to normalize for differences in the efficiency of transfection. Gancyclovir resistant clones were screened for *attR* sites. Two experiments were carried out and the bars reflect the means of the normalized activities (Additional file 1: Table S5). The analyses were carried out twice on a single *attP* array CCAG HyTK *attB* array reporter cell line and thus the data is shown as the mean of the two experiments. **C**; HT1080 cells that had been transfected with the empty expression vector (CCAG iresZeo) or with the indicated integrase expression vector were transiently transfected with the *attP* array CCAG HyTK *attB* array reporter and scored for site-specific recombination by PCR. For φ370 and TG1 integrases the integrase expression vector was co-transfected with the reporter construct, the remaining experiments were carried out with HT1080 cell lines that had been stably transfected with the respective integrase expression construct.

We therefore used electroporation to transfect linearized integrase expression constructs into each of the two independent cell lines containing the *attP* array CCAG HyTK *attB* array reporter used in the transient expression experiments and selected for clones containing stably integrated, integrase expression constructs (Additional file 1: Table S4). The yield of clones generated by the integrase expression constructs was at least an order of magnitude lower than those recovered following transfection with the empty expression vector suggesting that all of the integrases were toxic to various degrees. The TG1 and φ370.1 integrases appeared particularly toxic by this criterion because these consistently gave us no stably transfected clones. We divided the clones that had been stably transfected with each of the integrases into two groups of approximately equal sizes. We used the first of these groups to estimate the ability of the integrases to promote site-specific recombination of the *attP* array CCAG HyTK *attB* array reporter construct within the first two weeks of exposure to integrase. In order to do this we applied gancyclovir selection to these clones and then assayed individual gancyclovir resistant clones for one of the recombinant products, *attR*, by PCR. The six integrases that had shown clearly detectable activity in the transient transfection experiment (R4, φC31, φBT1, Bxb1, SPBc and Wβ) showed activity after stable expression of the integrase (Figure 4A). The remaining integrases showed no evidence for site-specific recombinase activity following gancyclovir selection. We wanted to determine how accurately the six active integrases were mediating site-specific recombination. We therefore sequenced at least 7 PCR products containing recombinant *attR* sites derived from each of the six active integrases and showed that, with the notable exception of the R4 integrase, all exclusively yielded products that were consistent with conservative site-specific recombination (Table 2). In the case of the R4 integrase only 2/9 PCR products contained intact *attR* sites, demonstrating that this enzyme seems particularly damage prone, at least in human cells.

**Table 2:** Accuracy of integrase mediated site-specific recombination in human HT1080 and mouse ES cells

**Integrase**	**Sequence**	**Integrations**	**Deletions**
**BxB1**	**HT1080**	**ES**	
*attB*	TCGGCCGGCTTGTCGACGACGGCGGTCTCCGTCGTCAGGATCATCCGGGC		
*attP*	GTCGTGGTTTGTCTGGTCAACCACCGCGGTCTCAGTGGTGTACGGTACAAACCCCGAC		
*attL*	TCGGCCGGCTTGTCGACGACGGCGGTCTCAGTGGTGTACGGTACAAACCCCGAC	3/3	
*attR*	GTCGTGGTTTGTCTGGTCAACCACCGCGGTCTCCGTCGTCAGGATCATCCGGGC	7/7	2/2
fC31			
*attB*	TGCGGGTGCCAGGGCGTGCCCTTGGGCTCCCCGGGCGCGTACTCC		
*attP*	GTGCCCCAACTGGGGTAACCTTTGAGTTCTCTCAGTTGGGGG		
*attL*	TGCGGGTGCCAGGGCGTGCCCTTGAGTTCTCTCAGTTGGGGG	3/3	
*attR*	TTGCCCCAACTGGGGTAACCTTTGGGCTCCCCGGGCGCGTACTCC	7/7	3/3
**R4**			
*attB*	GCGCCCAAGTTGCCCATGACCATGCCGAAGCAGTGGTAGAAGGGCACCGGCAGACAC		
*attP*	AGGCATGTTCCCCAAAGCGATACCACTTGAAGCAGTGGTACTGCTTGTGGGTACACTCTGC		
*attL*	GCGCCCAAGTTGCCCATGACCATGCCGAAGCAGTGGTACTGCTTGTGGGTACACTCTGC	3/3	
*attR*	AGGCATGTTCCCCAAAGCGATACCACTTGAAGCAGTGGTAGAAGGGCACCGGCAGACAC	2/9	1/2
del clone 1 DattR	AGGCATGTTCCCCAAAGCGATACCAC AAGCAGTGGTAGAAGGGCACCGGCAGACAC		
del clone 2 DattR	AGGCATGTTCCCCAAAGCGATACCACTT AAGCAGTGGTAGAAGGGCACCGGCAGACAC		
del clone 3 DattR	AGGCATGTTCCCCAAAGCGATACCACTTG GCAGTGGTAGAAGGGCACCGGCAGACAC		
del clone 4 DattR	AGGCATGTTCCCCAAAGCGATA GCAGTGGTAGAAGGGCACCGGCAGACAC		
del clone 5 DattR	AGGCATGTTCCCCAAAGCGATACCA GTGGTAGAAGGGCACCGGCAGACAC		
del clone 6 DattR	AGGCATGTTCCCCAAAGCGATACCA GAAGCAGTGGTAGAAGGGCACCGGCAGACAC		
del clone 7 DattR	AGGCATGTTCCCCAAAGCGATACCAC AAGCAGTGGTAGAAGGGCACCGGCAGACAC		
ES del clone 1 DattR	AGGCATGTTCCCCAAAGCGATACCA AGCAGTGGTAGAAGGGCACCGGCAGACAC	1/2	
intn clone 1-3 DattR	AGGCATGTTCCCCAAAGCGATA CAGTGGTAGAAGGGCACCGGCAGACAC	3/3	
fBT1			
*attB*	GTCCTTGACCAGGTTTTTGACGAAAGTGATCCAGATGATCCAGCTCCACACCCCGAACGC		
*attP*	GGTGCTGGGTTGTTGTCTCTGGACAGTGATCCATGGGAAACTACTCAGCACCACCAATG		
*attL*	GTCCTTGACCAGGTTTTTGACGAAAGTGATCCATGGGAAACTACTCAGCACCACCAATG	3/3	
*attR*	GGTGCTGGGTTGTTGTCTCTGGACAGTGATCCAGATGATCCAGCTCCACACCCCGAACGC	7/7	2/2
intn clone 1DattR	GGTGCTGGGTTGTTGTCTC GATCCAGATGATCCAGCTCCACACCCCGAACGC	1/3	
**Wβ**			
*attB*	AAGGTAGCGTCAACGATAGGTGTAACTGTCGTGTTTGTAACGGTACTTCCAACAGCTGGCG		
*attP*	TAGTTTTAAAGTTGGTTATTAGTTACTGTGATATTTATCACGGTACCCAATAACCAATGAA		
*attR*	TAGTTTTAAAGTTGGTTATTAGTTACTGTGATATTTGTAACGGTACTTCCAACAGCTGGCG	7/7	2/2
**SPBc**			
*attB*	AGTGCAGCATGTCATTAATATCAGTACAGATAAAGCTGTATCTCCTGTGAACACAATGGGTG	7/7	2/4
*attP*	AAAGTAGTAAGTATCTTAAAAAACAGATAAAGCTGTATATTAAGATACTTACTAC		
*attR*	AAAGTAGTAAGTATCTTAAAAAACAGATAAAGCTGTATCTCCTGTGAACACAATGGGTG		
ES del clone 1 DattR	AAAGTAGTAAGTATCTTAAAAAACA———AGCTGTATCTCCTGTGAACACAATGGGTG	1/4	
ES del clone 2 DattR	*aaaatacagcgtttttcatgtacaactatact*GCTGTATCTCCTGTGAACACAATGGGTG	1/4	
**TP901**			
*attB*	TGATAATTGCCAACACAATTAACATCTCAATCAAGGTAAATGCTTTTTCGTTTT		
*attP*	AATTGCGAGTTTTTATTTCGTTTATTTCAATTAAGGTAACTAAAAAACTCCTTT		
*attR*	AATTGCGAGTTTTTATTTCGTTTATTTCAAGGTAAATGCTTTTTCGTTTT	4/5	
del clone 1 DattR	AATTGCGAGTTTTTATTTCGTTTATTTC GGTAAATGCTTTTTCGTTTT	1/5	

The results show the sequences of the recombinant sites detected following integrase mediate site specific recombination in human HT1080 or mouse ES cells. Underlined and in lowercase in the case of one sequence from a deletion event occurring in ES cells following expression of the SPBc integrase is the sequence of a flanking **φ**370.1 *attP* site discussed in the text.

We wanted to know whether the complete failure to detect deletion activity after two weeks of growth in the presence TP901-1, FC1, φ370.1, K38, φRV, A118, BL3 and MR11 integrases was because these recombinases were slow or because they were damaging the target sites. We therefore applied hygromycin selection to the second group of clones that had been stably transfected with the integrase expression construct but not exposed to gancyclovir, then relaxed hygromycin selection and analysed the clones for any detectable recombination after a further two weeks by PCR. The results were clear; TP901-1 integrase showed detectable recombination after further culture indicating that it was indeed slow but the remaining integrases showed no evidence of recombination (Additional file 1: Table S4). As before we determined the sequence of the PCR products containing the predicted *attR* site generated by the TP901-1 integrase; 4 were intact *attR* sites and 1 was damaged thus showing that TP901-1 integrase is like the R4 integrase and prone to site damage. We wanted to know whether the remaining 8 integrases that had failed to show productive recombination were attempting recombination but were in fact damaging the attachment sites. We therefore sequenced their substrate attachment sites (*attP* and *attB*) in the integrated reporter plasmids in two of the hygromycin resistant clones that had been transfected with the respective integrase expression constructs. None showed evidence of site damage.

Thus we concluded that the R4, φC31, φBT1, Bxb1, SPBc, TP901-1 and Wβ integrases are active on substrates integrated in to the genome of HT1080 cells although the TP901-1 integrase promotes recombination slowly and that the FC1, φK38, RV, A118, BL3 and MR11 integrases are not detectably active on integrated substrates. We cannot make any statement about the TG1 and φ370.1 integrases because we were unable to recover clones expressing these integrases for experimental analysis. We attempted to use western blotting to assay for expression of the integrases using with an antibody to the N-terminal StrepII tag but we were unable to detect any signal with any of the integrases suggesting that they are all expressed at low levels. Rank ordering of the deletion activities of the integrases in this experiment indicated the following Bxb1 = φC31 = φBT1 > R4 = Wβ > SPBc > TP901-1. The utility of the R4 integrase would seem to be limited by its liability to site damage. The purpose of this part of our project was to identify those enzymes that were active in vertebrate cells and we did not investigate the background of GANC^r^*attR*^*-*^ clones. Although such clones were seen in the cells transfected with the empty vector and may arise from background silencing of the *attP* CCAG HyTK *attB* indicator gene or from loss of the chromosome carrying this gene, they occur at a higher level in the clones that had been transfected with integrases (Additional file 1: Table S4). We cannot exclude the possibility that they arise as a result of recombinase-mediated target site damage, although the accurate recombination activities seen with five of the seven active integrases would suggest that this is unlikely. The source of these background clones is therefore unclear.

It is also clear that not all of the clones that were successfully transfected with a construct expressing an active integrase yielded recombinant products. Thus even with the Bxb1 and φC31 integrases about 30% of the clones that were resistant to the antibiotic used to select for the presence of the expression construct failed to yield clones that were resistant to gancyclovir and contained an *attR* site. One hypothesis was that these clones failed to express sufficient integrase to bind and synapse the substrates, processes that are dependent on the affinity of integrase for its *attP* and *attB* sites and the expression level of integrase. We tried to test this idea by using the StrepII epitope with which we had tagged all of the integrases but, as before, were unable to do so for all of the integrases because the StrepII epitope tag was insufficiently sensitive and no signal was obtained in the western blots. However we had specific polyclonal antibodies for the φC31 and φBT1 integrases that we expected would be more sensitive and indeed they allowed us to measure the presence of the respective integrases by western blotting,. The results of this analysis was consistent with the notion that at least for some integrases the failure of site-specific recombination was associated with inadequate or low levels of expression (Additional file 1: Figure S2). The success of the western blotting using the polyclonal antibodies also demonstrates that the previous failure to detect integrase expression in the human cells using the StrepII tag and antibody was due to the relatively low sensitivity of this system.

The results obtained with the human HT1080 cells posed the question of whether they were generally true for vertebrate cells. Genome engineering of mouse embryonic stem cells (ES cells) is widely practiced and so we used a deletion strategy to assay the activities of the integrases in mouse ES cells. We used sequence targeting to introduce the *attP* array CCAG HyTK *attB* array deletion reporter cassette into the ROSA26 locus (Additional file 1: Figure S1) and then assayed for deletion by gancyclovir resistance following transient transfection with the set of integrase expression constructs described above. In order to avoid problems with toxicity that would compromise the practical significance of any results we chose to use transient assays and an internal control to carry out the experiment. In this ES cell system there appeared to be a clearer difference between the numbers of gancyclovir-resistant clones seen with the empty vector and those with expressing integrase than was observed in the HT1080 cells. The difference was not absolute however and identification of active integrases also required PCR analysis for the presence of a recombinant *attR* site. The results (Additional file 1: Table S5 and Figure 4B) were similar but not identical to those seen in the HT1080 cells; R4, φC31, φBT1, Bxb1, SPBc, Wβ and TG1 integrases showed detectable activity but the TP901-1 integrase did not. The activity seen with the TG1 integrase was, however, weaker than seen with the others with only one out of eight clones containing a detectable *attR* site. Rank ordering of the deletion activities of the integrases in this experiment indicated the following Wβ > Bxb1 > φC31 > SPBc > R4 > φBT1. We analyzed the accuracy of the site-specific recombination mediating the deletion reaction in two gancyclovir-resistant clones generated by the Bxb1, R4, Wβ and SPBc integrases. These results demonstrated that the Bxb 1, φBT1 and Wβ integrases all mediated site-specific recombination accurately but that the R4 integrases was associated with a deletion in one of the two recombination products analyzed and that both of the SPBc products were deleted. In the case of the SPBc integrase the region deleted 171 bp in the *attP* array extending as far as the φ370.1 *attP* site raising the possibility of a lack of specificity in *attP* site recognition by the integrase. This however seems unlikely because the breakpoint in the φ370.1 *attP* site is not at the proposed recombination junction but 6 bp 3′ of the point of symmetry defining the pseudo-palindrome of this attachment site.

The failure to detect recombinase activity mediated by the φ370, FC1, TG1, RV, FC1, φK38, MR11, A118 and BL3 integrases in HT1080 cells using deletion substrates integrated into the vertebrate genome posed the question as to whether these integrases were active in HT1080 cells or whether they were simply prevented from acting by the fact that the target sites were integrated into the genome. We therefore carried out a series of transient tranfection experiments in which the deletion substrate plasmid was transiently transfected into HT1080 cells that had been stably transfected with an expression plasmid for one of these integrases or, in the case of the φ370 and TG1 integrases,(where such stably transfected cell lines did not exist) co-transfected the respective expression plasmid and deletion substrate plasmid and then analyzed the extracted DNA for deleted plasmid after 72 hours by PCR. The recombinants were assayed using one of two PCR reactions for which the Bxb 1 or Wβ integrases acted as positive controls. The results (Figure 4C) demonstrate that the RV, φK38, MR11, A118 and BL3 integrases were in fact active in the HT1080 cells and thus we conclude that these integrases are unable to promote site-specific recombination when their substrates are integrated into the genome of the HT1080 cells but can do so when they are present extra-chromosomally. We can make no statement about the φ370, FC1, TG1 integrases as we have no evidence to determine whether they are expressed.

### Comparative integration activities of seven unidirectional phage integrases in mammalian cells assayed by recombinase mediated cassette exchange

**Figure 5:**
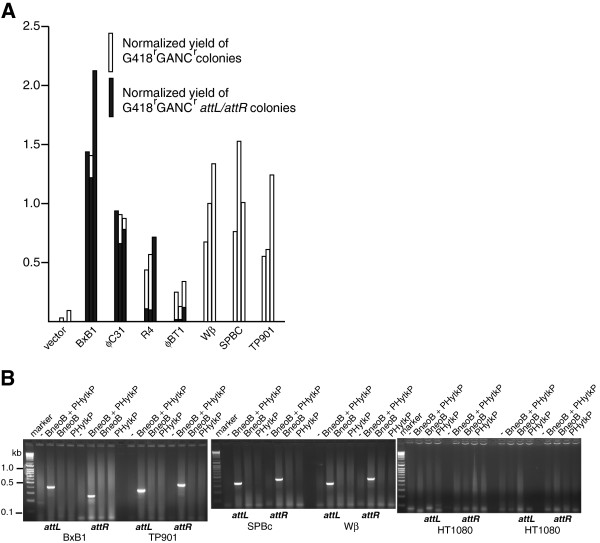
**Comparing the activity of seven different serine recombinases in human HT1080 cells for their utility in recombinase mediated cassette exchange. A**. Cell lines containing a single integrated *attP* array CCAG HyTK *attP* array reporter construct and stably expressing the indicated integrase were transfected with the *attB* array CCAG neo *attB* array integration reporter construct using lipofectamine. The experiment was carried out three times using two independent cell lines for each of the seven integrases. The number of G418-resistant clones generated by the cassette exchange reaction promoted by the different integrases was normalized by transfection using lipofectamine with a uniform amount of linearized CCAG neo plasmid. Open bars correspond to the number of colonies generated with the reporter plasmid divided by the number of colonies generated with the linearized CCAG neo (for the raw data see Additional file 1: Table S6). Between seven and ten colonies of each transfection were picked and assayed for site-specific recombination by PCR and the total yield of colonies generated by site-specific recombination is represented by the filled bars. **B**. Cell lines that had been stably transfected with the indicated integrase expression construct were transiently transfected with the indicated integration reporter construct and after three days assayed for site specific recombination by PCR. Two different reactions were used for the assay; one for the Bxb1 integrase and the other for the remaining integrases. The gel on the final panel shows the PCR reaction products obtained when the indicated reporter constructs were transfected into HT1080 cells that expressed no integrase and assayed for site-specific recombination by either of the two reactions.

Site-specific integration, and in particular the exchange of marker cassettes, collectively termed recombinase mediated cassette exchange, is an important technique for the precise introduction of DNA into cells, in particular for the comparative analysis of different genes integrated at the same docking site. The uni-directional serine integrases are ideally suited to this application as they require only simple attachment sites and no host accessory proteins. We wanted to determine which of the seven integrases that we had identified above as being active in promoting site-specific deletion in human cells, also functioned in promoting site-specific cassette exchange. Our previous experiments had shown the merits of using cell lines that contained arrays of attachment sites that were then stably transfected with different integrase-expressing plasmids as reagents for accurate comparisons of integrase activity. We first introduced an integration target*; attP* array CCAG HyTK *attP* array expressing the hygromycin-thymidine kinase fusion flanked by arrays of *attP* sites into human HT1080 cells by electroporation and, as before, selected structurally intact, single copy integrants. We then transfected these cells with individual integrase expression plasmids for each of the seven integrases that had been shown to be active in the deletion assay and again selected for stable integrants. In order to compare the cassette exchange activities of the different integrases we then transiently transfected two completely independent clones with the integration substrate, *attB* array CCAG Neo *attB* array (Figure 2). The work described above indicated that the integrases were toxic to varying degrees (Additional file 1: Table S4) and so we expected that there would be different numbers of resistant clones derived from the transfection experiments of clones expressing different integrases, despite similar amounts of plasmid being used in the transfection. We controlled for such differences by transfecting the integrase-expressing clones with a linearized CCAG Neo plasmid in a parallel experiment carried out at the same time as they were transfected with the circular *attB* array CCAG Neo *attB* array plasmid*.* We selected for G418 resistance in both cases and normalized the experimental transfections using the yield of clones generated following transfection with the linearized CCAG Neo. We then assayed for cassette exchange in the experimental clones by selecting the G418 resistant clones for gancyclovir resistance to identify those clones that had lost the CCAG HyTK marker and by PCR in order to identify the *attL* and *attR* products of a site-specific recombination reaction. We carried out three such assays on two independent *attP* CCAG HyTK *attP,* integrase-expressing clones. The results (Additional file 1: Table S6, Figure 5A) of these experiments revealed consistent and significant differences between the seven integrases in terms of their abilities to mediate site-specific integration. At one extreme were the Bxb1 and φC31 integrases which promoted efficient and accurate site specific integration and at the other were the three integrases Wβ, SPBc and TP901-1 which generated clones that had the phenotypes expected of cassette exchange through site-specific recombination i.e. they were gancyclocvir -resistant and G418-resistant, but for which we could not obtain PCR products for *attL* or *attR*. We sequenced the *attL* and *attR* sites generated by site-specific integration in the Bxb1, φC31, R4 and φBT1 integrase-expressing clones. The Bxb1 and φC31 integrases products were as predicted but in three cases the *attR* product generated by the R4 integrase was damaged and similarly one of the three *attR* sites generated by the φBT1 integrase was also damaged. The experimental results shown in Additional file 1: Table S6 could be interpreted to suggest that there is a high background of non-specific integration of the circular plasmids into the genome of the HT1080 cells in the absence of any integrase. Such an interpretation would only be valid however if it the cells containing the empty expression vector were transfected with equal efficiency to those expressing any of the integrases. Given the toxicity of the integrases seen in the results shown in Additional file 1: Table S4 this is almost certainly not the case and thus the relatively apparently high background of non-specific integration simply reflects the fact that the cells that do not express integrase are just more easily transfected than those that do.

**Figure 6:**
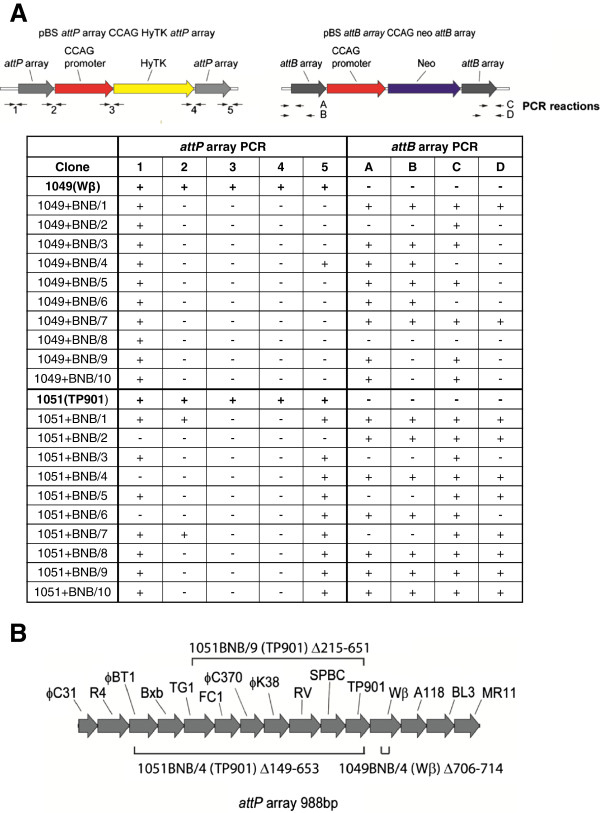
**Analysis of G418-resistant, gancyclovir-resistant, *attR***^***-***^***, attL***^***-***^**clones generated by the Wβ and TP901 integrases. A**. Ten G418-resistant, gancyclovir-resistant, *attR*^*-*^*, attL*^*-*^ clones generated by one or other of these two integrases were analysed by PCR across the indicated sequences in each of the two reporter plasmids used in the transfection. The numbers below the plasmid maps indicate the PCR reactions assayed in the table. Primer sequences used for these assays are listed in the (Additional file 1: Table S7). **B**. Sequence analysis of damaged sites in the three indicated clones. The diagram shows the regions deleted in the remnant of the flanking arrays found in each of three clones.

We wanted to understand how the gancyclocvir-resistant, G418-resistant, *attL*^*-*^, *attR*^*-*^ clones had been generated by the Wβ, SPBC and TP901-1 integrases. First of all we wanted to confirm that indeed these integrases were active in the human cells and in order to investigate this we assayed extra-chromosomal site-specific recombination following transient transfection. We therefore took one of the clones expressing each of these integrases and Bxb1 integrase as a control and then transiently transfected the cells with the integration substrate plasmids either alone or together and then confirmed site-specific recombination by PCR for each of the respective *attR* and *attL* sites (Figure 5B). We supposed that the gancyclocvir-resistant, G418-resistant, *attL*^*-*^, *attR*^*-*^ clones generated by these recombinases arose as a result of DNA damage arising as a result of abortive recombination. We therefore characterized the structure of the remains of the docking site (the *attP* array CCAG HyTK *attP* array plasmid) and the integrated *attB* array CCAGNeo *attB* array plasmid in 10 clones derived by transfection of either the Wβ or TP901-1 integrase expressing clones (Figure 6A). This analysis showed evidence of target site damage with extensive deletion of the HyTK coding region and flanking sequences. In the case of the TP901-1 integrase six of the clones (numbers 3, 5, 7, 8, 9 and 10) the internal sequences (2, 3 and 4 in Figure 6A) were deleted but flanking sequences (1 and 5 in Figure 6A) were intact suggesting the possibility that the two *attP* sites had simply ligated together after deletion of the intervening DNA. We therefore analysed the residual *attP* sequences in three clones: two, numbers 4 and 9, showed evidence of deletion of the residual *attP* sequences (Figure 6B) while in a third, number 8, the residual *attP* sequence was intact. For both integrases the incoming *attB* array CCAG Neo *attB* array plasmid also showed evidence of DNA damage, but in this case the damage was associated with the DNA flanking the cognate attachment sites. Thus in eight out of ten of the Wβ derived clones one or other of the attachment sites showed evidence of damage and three out of the ten TP901-1 derived clones showed evidence of damage to one or other of the attachment sites. The conclusion that we draw from these studies is that DNA damage is limiting the ability of the Wβ, SPBc and TP901-1 integrases to mediate site-specific recombination and cassette exchange in vertebrate cells and compromises the potential utility of the R4 and φBT1 integrases. However we are able to rank the utility of the other four enzymes: Bxb1 > φC31 > R4 > φBT1.

As before we also tried to assay the ability of these seven enzymes to mediate site-specific integration in mouse cells using transient expression of the integrase. None were detectably active suggesting that as in human cells detectable integration with this configuration of attachment sites requires stable expression of the integrase.

## Conclusions

The major conclusion following from this work is that although all of the fifteen unidirectional serine integrases for which both attachment sites have been identified are active in *E.coli*, only four of these; Bxb1, φC31, R4 and φBT1 are able to mediate accurate site-specific integration into genomic DNA in human cells and rank such that Bxb1 is marginally better than φC31 and both are better than R4 and φBT1 integrases. The three integrases from the phages Wβ, SPBc, TP901-1 are active in vertebrate cells as judged by their ability to mediate site-specific deletion and by their ability to mediate integration reactions extra-chromosomally but fail to complete successful site-specific integration when attempting an integration by cassette exchange because they damage one or other of the participating DNA molecules. Although there are differences (Figure 4) between the activities seen with the different integrases in human and mouse cells the data the overall pattern of activites in the two cell types is such that it would seem prudent to adopt the B×b 1 integrase as the first choice in both. We have not investigated the causes of the different activities but they must reflect interactions between the respective integrases and host encoded proteins.

Our observations have three practical implications and pose one question. The practical implications are first that the B×b 1 integrase should be the first choice for any genome engineering in vertebrate cells that requires the use of a serine integrase. Second, that screening for more serine integrases that can be used in vertebrate cells is likely to have a low success rate and, third, that it is necessary that all site-specific integrants, particularly those generated by the R4 and φBT1 integrases should be checked for the fidelity of the recombination reaction.

**Figure 7:**
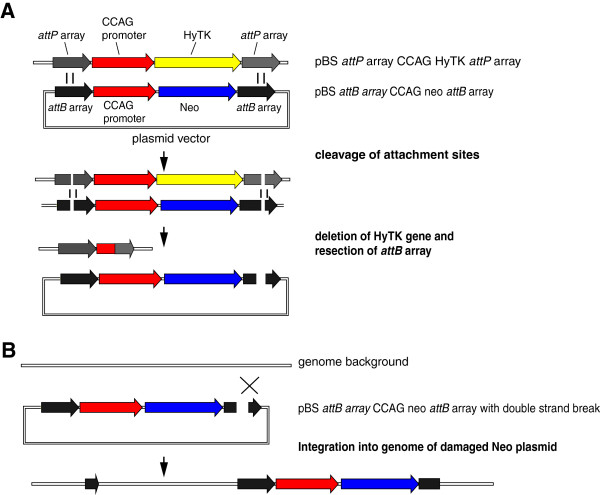
**Hypothetical mechanism for the origin of the G418**^**r **^**gancyclovir**^**r**^**, *attR***^***-***^***, attL***^***-***^**clones generated by the serine integrases during integration reactions.** The figure shows a two step model for the origin of the GANC^r^ Neo^r ^*attL*^*- *^*attR*^*-*^clones arising during the integration experiments. **A** illustrates the first step in the model; the deletion of the HyTK gene and **B** illustrates the second step; the integration of the CCAGneo gene. Further details are discussed in the text.

The results obtained in the integration experiments with the Wβ, SPBc, TP901-1 integrases pose the question of how such damage arises. One possible mechanism is set out in Figure 7. In this schema one or other of the attachment sites on the incoming donor plasmid, *attB* array CCAG neo *attB* array forms a synaptic complex with an *attP* site in the integrated docking cassette located on *attP* CCAG HyTK *attP*. The *attP* and *attB* sites are then cleaved but because the two participating sites are embedded in chromatin, strand exchange and/or re-joining of the DNA backbone are inhibited. Possibly the intermediate complex, containing the covalent attached integrase subunits to the cleaved DNA, is unstable and dissociates, or the process of strand exchange by subunit rotation is interrupted. The cleaved DNA covalently linked to integrase subunits leads to resection of the target *attP* site by DNA repair pathways which in turn leads to loss of the counter-selectable HyTK gene (Figure 7A). The concomitant double strand break in the *attB* array CCAG neo *attB* array plasmid enables this plasmid to integrate efficiently elsewhere in the genome (Figure 7B). However not all of the gancyclovir-resistant, G418-resistant, *attL*^*-*^, *attR*^*-*^ clones recovered following transfection of the Wβ or TP901-1 integrase expressing lines showed evidence of damage at one or other of the donor *attB* sites and one assumes that these clones arise by a similar but more complicated mechanism in which the transfected cell takes up more than one of the these plasmids and that it is one of the plasmids that have not participated in the abortive attempt at site specific integration that integrates into the host cell genome.

If chromatin is inhibiting strand exchange by the serine integrases, one might also have reasonably expected chromatin to alter other activities of integrase such as site-selection. *In vitro* φC31 integrase only recombines *attP × attB* and is never active on other pairs of attachment sites including *attP* with *attP* or *attB* with *attB*. This site-selectivity is explained as only integrase dimers bound to *attP* and to *attB* can tetramerise to form the synaptic complex. Within this complex activation of DNA cleavage occurs. In all of our experiments we did not observed altered site-selectivity or DNA damage arising from some attempt at recombination of two *attP* sites or two *attB* sites. The absence of any change in site-selectivity in eukaryotic chromatin implies that the proposed conformational differences between integrase bound to an *attP* site and to an *attB* site are robust to withstand any fortuitous protein-protein interactions arising with chromatin.

The differences seen between the activities of the integrases in *E. coli* and in vertebrate cells on extra-chromosomal substrates on one hand and on substrates integrated into the genome on the other and the explanation for the site damage seen in the integration reactions both suggest that chromatin and other DNA binding proteins are important factors in limiting the activity of site-specific recombinases in vertebrate cells. It may therefore be of value in future experiments to determine how the activity of the integrases vary according to the position of the target sites in the genome.

## Methods

### Bacterial strains, plasmids and molecular biology

*E. coli* K12 DH5α was used as a general cloning host and propagated on LB medium containing appropriate supplements or antibiotics (ampicillin (100 μg/ml), chloramphenicol (50 μg/ml), X-gal (120 μg/ml), IPTG (40 μg/ml). *E. coli* BL21 (DE3) was used as a protein over-expression host. To construct the integrase expression plasmids, the integrase genes were amplified by PCR from either phage templates or from plasmids and inserted into pET21a vector (Novagen) using either the In-Fusion cloning system (Clontech) or T4 ligase and compatible restriction sites. The native sequences were used as the templates for the amplification of all integrase genes except for φC31, A118, FC1 and φK38 for which templates were derived from synthetic, codon optimised forms (Genescript). Each integrase gene was modified to include a StrepII tag at the 5′ end and a nuclear localisation sequence (NLS) at the 3′ end. Reporter plasmids containing cognate *attP* and *attB* sites for all the integrases were cloned into pACYC184. PCR was used to amplify the *lacZα* gene using forward and reverse primers that contained the *attB* and *attP* sites (both approximately 50 bp in length) in head-to-tail orientation (Additional file 1: Table S1). All of the constructed plasmids were verified by sequencing (Dundee Sequencing Service). The sequences of all PCR plasmids and primers used in the bacterial work are listed in Additional file 1: Table S1 and Additional file 1: Table S2.

### Recombination assays in *E. coli*

The activities of the cloned integrases in *E. coli* were assessed by two assays, which relied on different expression regimes for the integrases. The expression vector pET21a has a T7 RNA polymerase promoter to drive the transcription of the integrase genes and this was used to ensure expression of the integrase genes in the *E. coli* host BL21(DE3). In the absence of T7 RNA polymerase, as is the case in *E. coli* DH5a, expression of integrase is dependent on the recognition of the T7 promoter by host RNA pol ymerase, which will result in lower expression.

DH5α cells containing the *attB/attP* reporter plasmids were transformed with the appropriate integrase expression plasmid and plated out on LB agar plates containing ampicillin (100 μg/ml), chloramphenicol (50 μg/ml), X-gal (120 μg/ml), IPTG (40 μg/ml) and incubated overnight at 37°C. Recombination between the *att* sites deletes the *lacZα* gene from the reporter plasmid leading to white colonies and therefore recombinants (white colonies) were scored amongst a background of non-recombinants (blue colonies). The transformation plates derived from several integrases gave rise to only blue or light blue colonies. Restreaking of these colonies was performed to determine if white colonies (containing recombinant reporter plasmids) could segregate.

BL21(DE3) cells containing the *attB*/*attP* reporter plasmids were transformed with the appropriate integrase expression plasmid and plated out on LB agar plates containing ampicillin and chloramphenicol. After an overnight incubation a single colony was picked and grown in 5 ml 2YT containing ampicillin and chloramphenicol for 5 h at 37°C. The cells were induced with IPTG and grown overnight at 20°C after which 1 ml of this culture was used to prepare plasmid and the pellet from 4 ml was used in Western blotting to estimate the expression of integrase. Plasmids extracted from the overnight culture were used to transform DH5α cells and colonies growing on plates containing chloramphenicol, X-gal and IPTG after overnight at 37°C were examined. The proportion of recombinant versus non-recombinant plasmids was scored from the proportion of white versus blue transformants. PCR was carried out on 5 individual colonies for each integrase used in the assay to amplify and sequence the *attL* sites, remaining in the recombinant plasmids. Cell pellets from the overnight cultures were resuspended in LB, incubated in SDS sample buffer and the proteins separated on a 4-12% SDS gel (Expedeon, UK). The integrase protein was detected by Western blotting using a monoclonal antibody against the StrepII tag conjugated to horseradish peroxidise (IBA, Germany). The amount of protein present was determined by comparing the intensities of the bands from the Western blotting to those from a dilution series of purified StrepII-tagged φC31 integrase. The intensity of the bands was determined using ImageJ (NIH) and the results were expressed as μg of total protein per ml culture.

### *In vitro* recombination assay

Integrase recombination activity was assayed *in vitro* using substrates in which the attachment sites were present either isolated from other attachment sites or within the arrays containing the attachment sites for all 15 integrases of interest. The combination of substrates was as follows: pRT702 (φC31 *attP* site) and pRT600 (φC31 *attB* site), pRT702 and pUC57_*attB* array, pUC57_*attP* array and pRT600, pUC57_*attP* array and pCCAG_*attB* array, pCCAG_*attP* array and pUC57_*attB* array. The substrates were incubated in recombination buffer (10 mM Tris pH 7.5, 100 mM NaCl, 5 mM DTT, 5 mM spermidine, 4.5% glycerol, 0.5 mg/ml BSA) and φC31 integrase (0 – 700 nM) for 1 h at 30°C [26] After heat inactivation at 80°C for 10 min the reaction was digested with HindIII or BamHI and the recombination molecules detected by gel electrophoresis. Gel images were analysed using ImageJ (NIH); the intensity of the bands was determined (after subtraction of base line intensities) and used to quantify the depletion of substrates and appearance of products.

### Human cell culture

Human HT1080 fibrosarcoma cells were grown as described previously except that RPMI 1640 rather than Dulbecco’s modified Eagles Medium was used as the un-supplemented medium. G418, Hygromycin and gancyclovir (Invitrogen) were used at 400 mg/ml, 100 mg/ml and 20 μM respectively for selection. Cells were transfected with DNA either by electroporation using a BTX ECM 630 at 400 V, 50 Ω and 250 μF or using Lipofectamine (Invitrogen) as recommended by the manufacturers. We used electroporation with 500 μg of linearized DNA and zeocin selection for the stable introduction of the integrase expression constructs and lipofection of 1.6 μg of closed circular DNA for the transient introduction of either substrate or integrase expression construct.

### ES cell manipulation and analysis

#### Targeting vector construction and gene targeting

The insertion of the *attP*-Hygro-TK-*attP* array at the ROSA26 locus was performed by gene targeting in E14tg2a ES cells (Additional file 1: Figure S1). A targeting vector for the ROSA26 locus, pRosa26.10 [27] was adapted by the insertion of an AscI-NsiI-SacII polylinker, made by oligonucleotide annealing, into the AscI and SacII sites 3′ and 5′ of the homology arms, creating plasmid pRosa26-PL. The final targeting vector, pRosa26-PHB containing the *attP*-Hygro-TK-*attB* arrays, was constructed by inserting the arrays from pBS*attP*CCAGHyTK*attB* into pROSA26-PL via the 5′ AscI and the 3′ NsiI sites.

The targeting vector was linearized by XhoI digestion and electroporated into 1×10^7^ E14tg2a cells at 500 V, 3 uF. Cells were plated on gelatin and recombinant clones were recovered by selection in Hygromycin 75 ug/ml. Targeted clones were identified by long range PCR screening using primers 5′-GGCACTACTGTGTTGGCGGA-3′ and 5′-GGCCAGCTTATCGATACCGT-3′ for the 5′ end; 5′-AGCGAGGGCTCAGTTGGGCTGTTT-3′ and 5′-CTCAGTGGCTCAACAACACTTGGTCA-3′ for the 3′ end. Single copy integration events were confirmed by Southern blotting using an EcoRV digest and an internal Hygromycin probe.

#### Transient assays of integrase-mediated deletion in ES cells

1 × 10^6^*attP*-Hygro-TK-*attB* ES cells were electroporated with 5 μg of the integrase vectors and 200 ng of a control plasmid containing a functional neomycin cassette to control for transfection efficiency. Electroporation was performed using the Neon transfection system (Invitrogen) (3 × 1400 V, 10 ms). Cells were plated on 2 wells of a 6 well plate and selected in either 3 μM Gancyclovir or 350 μg/ul G418. On the 8^th^ day of selection resistant colonies were stained in methylene blue and counted. Deletion efficiencies were calculated from the number of gancycolvir resistant colonies normalised against transfection efficiency by assessing the number of G418 resistant clones in the replica plating. All comparison transfection experiments were repeated twice using 2 independently targeted ES cell clones.

Individual gancyclovir resistant colonies were picked and expanded for the preparation of genomic DNA. PCR analysis using primers del-Rosa-F1 (5′ GATCGAGGTGCCCCAACTGGGGTAACCT TT-3′) and del-Rosa-R1 (5′-CCGCGGATGCATAGCGGATAACAATTTCAC-3′) which bind at the very 5′ and 3′ of the integrated *attP*-Hygro-TK-*attB* array and amplify across the expected deletion events (Additional file 1: Figure S1), was performed to confirm the deletion event had occurred. PCR products were sequences to assess the nature and position of the *attP x attB* recombination and the sequences of the resulting *attR* sites. Gancyclovir resistant clones that failed to yield an amplicon using this PCR strategy were further investigated for aberrant recombination events using primers which amplify the hygromycin sequence (5′- GAAGAATCTCGTGCTTTCAGCTTCGATG-3′; 5′- AATGACCGCTGTTATGCGGCCATTG-3′) and the thymidine kinase sequence (5′-TCTGGACCGATGGCTG TGTA-3′; 5′-AGACGTGCATGGAACGGAGG-3′).

## Competing interests

The authors declare that they have no competing interests.

## Authors’ contributions

Zy X, LT and BD carried out experimental work, WB, MS and BD designed the experiments, RC conceived the project. All authors read and approved the final manuscript.

## Supplementary Material

Additional file 1: Table S1 Plasmids used to assay integrase activity in *E.coli, ***Table S2.** Primers used to construct expression plasmids and assay integrase activity in *E.coli,***Table S3.** The results of two transient transfection experiments in which the integrase shown in the first column was introduced into each of two cell lines containing a single copy of the deletion assay reporter plasmid located intact at a single site in the genome of HT1080 cells. **Table S3.** Assaying integrase activity in human HT1080 cells by deletion activity following transient transfection of integrase expression plasmid, **Table S4.** Assaying integrase activity in human HT1080 cells by deletion activity following genomic integration of integrase expression plasmid, **Table S5.** Assaying integrase activity in mouse ES cells by deletion activity following transient transfection of integrase expression plasmid, **Table S6.** Assaying site-specific integration activity of seven integrases able in human HT1080 cells, **Table S7.** Primers used to analyse re-arrangements associated with cassette exchange integrations: illustrated in Figure 6 of main text, **Figure S1.(A)** Targeting the ROSA26 locus in mouse ES cells with a deletion reporter construct. **(B)** the targeted locus before deletion **(C)** the targeted locus after deletion. Sequences of the Del-Rosa primers are in the main text, **Figure S2.** western blot analysis of φC31 and φBT1 integrase expression in HT1080 clones that contain a single integrated copy of the *attP* array CCAG HyTk *attB* array reporter and have been stably transfected with an integrase expression plasmid but fail to delete the HyTk gene. Click here for file
