# Supplementary material for: Accuracy and efficiency define Bxb1 integrase as the best of fifteen candidate serine recombinases for the integration of DNA into the human genome
Source: BMC Biotechnol. 2013 Oct 20;13:87. doi: 10.1186/1472-6750-13-87 (PMC4015280; doi:10.1186/1472-6750-13-87)
Supplement: Additional file 1: Table S1 — Plasmids used to assay integrase activity in E.coli, Table S2. Primers used to construct expression plasmids and assay integrase activity in E.coli,Table S3. The results of two transient transfection experiments in which the integrase shown in the first column was introduced into each of two cell lines containing a single copy of the deletion assay reporter plasmid located intact at a single site in the genome of HT1080 cells. Table S3. Assaying integrase activity in human HT1080 cells by deletion activity following transient transfection of integrase expression plasmid, Table S4. Assaying integrase activity in human HT1080 cells by deletion activity following genomic integration of integrase expression plasmid, Table S5. Assaying integrase activity in mouse ES cells by deletion activity following transient transfection of integrase expression plasmid, Table S6. Assaying site-specific integration activity of seven integrases able in human HT1080 cells, Table S7. Primers used to analyse re-arrangements associated with cassette exchange integrations: illustrated in Figure 6 of main text, Figure S1.(A) Targeting the ROSA26 locus in mouse ES cells with a deletion reporter construct. (B) the targeted locus before deletion (C) the targeted locus after deletion. Sequences of the Del-Rosa primers are in the main text, Figure S2. western blot analysis of φC31 and φBT1 integrase expression in HT1080 clones that contain a single integrated copy of the attP array CCAG HyTk attB array reporter and have been stably transfected with an integrase expression plasmid but fail to delete the HyTk gene. [file 1472-6750-13-87-S1.docx]

**Xu et al Additional file**

**Table S1 Plasmids used to assay integrase activity in *E.coli***

| **Plasmid** | **Function** | **Vector** | **Primers** | **Reference** |
| --- | --- | --- | --- | --- |
| pLT01 | Expression of ϕC31 integrase | pET21a | LT01, LT02 | This work |
| pLT04 | Expression of ϕRV integrase | pET21a | LT06, LT07 | This work |
| pLT05 | Expression of TG1 integrase | pET21a | LT04, LT05 | This work |
| pLT07 | Expression of ϕBT1 integrase | pET21a | LT10, LT11 | This work |
| pLT08 | Expression of Wβ integrase | pET21a | LT08, LT09 | This work |
| pLT09 | Expression of Bxb1 integrase | pET21a | LT12, LT13 | This work |
| pLT11 | Expression of TP901 integrase | pET21a | LT14, LT15 | This work |
| pLT12 | Expression of R4 integrase | pET21a | LT56, LT57 | This work |
| pLT13 | Expression of BL3 integrase | pET21a | LT54, LT55 | This work |
| pLT14 | Expression of A118 integrase | pET21a | LT44, LT45 | This work |
| pLT17 | Expression of SPBc integrase | pET21a | LT52, LT53 | This work |
| pLT18 | Expression of MR11 integrase | pET21a | LT50, LT51 | This work |
| pLT20 | Expression of ϕ370.1 integrase | pET21a | LT46, LT47 | This work |
| pLT21 | Expression of K38 integrase | pET21a | LT48, LT49 | This work |
| pLT22 | Expression of FC1 integrase | pET21a | LT58, LT59 | This work |
| pLT25 | Bxb1 Recombination reporter plasmid; *attB- lacZ-attP* | pACYC184 | LT90, LT91 | This work |
| pLT27 | ϕC31 Recombination reporter plasmid; *attB- lacZ-attP* | pACYC184 | LT84, LT85 | This work |
| pLT29 | ϕBT1 Recombination reporter plasmid; *attB- lacZ-attP* | pACYC184 | LT86, LT87 | This work |
| pLT31 | MR11 Recombination reporter plasmid; *attB- lacZ-attP* | pACYC184 | LT92, LT93 | This work |
| pLT33 | R4 Recombination reporter plasmid; *attB- lacZ-attP* | pACYC184 | LT88, LT89 | This work |
| pLT34 | ϕ370.1 Recombination reporter plasmid; *attB- lacZ-attP* | pACYC184 | LT101, LT102 | This work |
| pLT37 | FC1 Recombination reporter plasmid; *attB- lacZ-attP* | pACYC184 | LT94, LT103 | This work |
|  | TG1 Recombination reporter plasmid; *attB- lacZ-attP* | pACYC184 |  |  |
|  | A118 Recombination reporter plasmid; *attB- lacZ-attP* | pACYC184 |  |  |
|  | Wβ Recombination reporter plasmid; *attB- lacZ-attP* | pACYC184 |  |  |
|  | ϕRV Recombination reporter plasmid; *attB- lacZ-attP* | pACYC184 |  |  |
|  | TP901 Recombination reporter plasmid; *attB- lacZ-attP* | pACYC184 |  |  |
|  | SPBc Recombination reporter plasmid; *attB- lacZ-attP* | pACYC184 |  |  |
|  | BL3 Recombination reporter plasmid; *attB- lacZ-attP* | pACYC184 |  |  |
|  | K38 Recombination reporter plasmid; *attB- lacZ-attP* | pACYC184 |  |  |
| pRT600 | ϕC31 Recombination reporter plasmid; *attB* | pGEM7 |  | [[1](#_ENREF_1)] |
| pRT602 | ϕC31 Recombination reporter plasmid; *attB* | pSP72 |  | [[1](#_ENREF_1)] |
| pRT702 | ϕC31 Recombination reporter plasmid; *attP* | pSP72 |  | [[1](#_ENREF_1)] |
| pUC57_attP array |  |  |  |  |
| pCCAG_attP array |  |  |  |  |
| pUC57_attB array |  |  |  |  |
| pCCAG_attB array |  |  |  |  |

1. Smith MCA, Till R, Smith MCM: Switching the polarity of a bacteriophage integration system. *Mol Microbiol* 2004, 51(6):1719-1728.

**Xu et al Additional file**

**Table S2 Primers used to construct expression plasmids and assay integrase activity in *E.coli***

| **Primer** | **Sequence** |
| --- | --- |
| LT01 | AAGGAGATATACATATGTGGTCCCACCCCCAGTTCGAGAAGATGGATACGTACGCTGGTGC |
| LT02 | GGTGGTGGTGCTCGAGCTACACCTTGCGCTTCTTCTTGGGCGCTGCGACGTCCTCGGTACC |
| LT06 | AAGGAGATATACATATGTGGTCCCACCCCCAGTTCGAGAAGATGAGATACACTACACCTGT |
| LT07 | GGTGGTGGTGCTCGAGCTACACCTTGCGCTTCTTCTTGGGTCGCCAATTCACCTGCACCCGTTCGGG |
| LT04 | AAGGAGATATACATATGTGGTCCCACCCCCAGTTCGAGAAGATGAGCGTGAAGGTTGAAGGC |
| LT05 | GGTGGTGGTGCTCGAGCTACACCTTGCGCTTCTTCTTGGGCGCCGCCGCTGTGAACCCGTT |
| LT10 | AAGGAGATATACATATGTGGTCCCACCCCCAGTTCGAGAAGATGTCGCCGTTCATCGCTCC |
| LT11 | GGTGGTGGTGCTCGAGCTACACCTTGCGCTTCTTCTTGGGCAGCGCCGCAAGCTCACGCT |
| LT08 | AAGGAGATATACATATGTGGTCCCACCCCCAGTTCGAGAAGATGAAATACGCTGTTTATGT |
| LT09 | GGTGGTGGTGCTCGAGCTACACCTTGCGCTTCTTCTTGGGCAGAGAAAATGTATATTCGAT |
| LT12 | AAGGAGATATACATATGTGGTCCCACCCCCAGTTCGAGAAGATGAGAGCCCTGGTAGTCAT |
| LT13 | GTGCGGCCGCAAGCTTCTACACCTTGCGCTTCTTCTTGGGCGACATCCCGGTGTGTAGCCG |
| LT14 | TATACATATGGCTAGCATGTGGTCCCACCCCCAGTTCGAGAAGATGACTAAGAAAGTAGCAAT |
| LT15 | GGTGGTGGTGCTCGAGCTACACCTTGCGCTTCTTCTTGGGAGCGAGTTGGAATTTAAATAT |
| LT56 | AAGGAGATATACATATGTGGTCCCACCCCCAGTTCGAGAAGATGAATCGAGGGGGGCCCAC |
| LT57 | GGTGGTGGTGCTCGAGCTACACCTTGCGCTTCTTCTTGGGCTCGGCCACGTCTCGCCACT |
| LT54 | TATACATATGGCTAGCATGTGGTCCCACCCCCAGTTCGAGAAGATGAAATTACGGGCTGCAAT |
| LT55 | GGTGGTGGTGCTCGAGCTACACCTTGCGCTTCTTCTTGGGTATGTTCCACTCTATTTTCA |
| LT44 | AAGGAGATATACATATGTGGTCCCACCCCCAGTTCGAGAAGATGAAGGCAGCTATTTATAT |
| LT45 | GGTGGTGGTGCTCGAGCTACACCTTGCGCTTCTTCTTGGGGAGCCATTCAATAGTAACTT |
| LT52 | AAGGAGATATACATATGTGGTCCCACCCCCAGTTCGAGAAGATGGAGTTAAAAAACATTGT |
| LT53 | GGTGGTGGTGCTCGAGCTACACCTTGCGCTTCTTCTTGGGGTGGAAACTATTAGTGGCTG |
| LT50 | AAGGAGATATACATATGTGGTCCCACCCCCAGTTCGAGAAGATGAAAGTAGCAATTTATAC |
| LT51 | GGTGGTGGTGCTCGAGCTACACCTTGCGCTTCTTCTTGGGATAAAAATCAATGTTTTTTA |
| LT46 | AAGGAGATATACATATGTGGTCCCACCCCCAGTTCGAGAAGATGAGAAAAGTAGCTATTTA |
| LT47 | GGTGGTGGTGCTCGAGCTACACCTTGCGCTTCTTCTTGGGGGCCAGCTGAAACTTAAAAA |
| LT48 | AAGGAGATATACATATGTGGTCCCACCCCCAGTTCGAGAAGATGTGGTCCCACCCCCAGTT |
| LT49 | GGTGGTGGTGCTCGAGCTACACCTTGCGCTTCTTCTTGGGCGTCCTCGCCGCCCATTTGA |
| LT58 | AAGGAGATATACATATGTGGTCCCACCCCCAGTTCGAGAAGATGAAGCGTGCAGCATTGTA |
| LT59 | GGTGGTGGTGCTCGAGCTACACCTTGCGCTTCTTCTTGGGAAATTTATATTTAATAATTA |
| LT90 | CCCGTCCTGTGGATCCTCGGCCGGCTTGTCGACGACGGCGGTCTCCGTCGTCAGGATCATCCGGGCGCGCAACGCAATTAATGTGAG |
| LT91 | CCGGCGTAGAGGATCCGGGTTTGTACCGTACACCACTGAGACCGCGGTGGTTGACCAGACAAACCATCATTGGGAAGGGCGATCGG |
| LT84 | CCCGTCCTGTGGATCCCGGTGCGGGTGCCAGGGCGTGCCCTTGGGCTCCCCGGGCGCGTACTCCACGCGAGTCAGT |
| LT85 | CCGGCGTAGAGGATCCCTACGCCCCCAACTGAGAGAACTCAAAGGTTACCCCAGTTGGGGCACTACAGGGATTTTG |
| LT86 | CCCGTCCTGTGGATCCGTCCTTGACCAGGTTTTTGACGAAAGTGATCCAGATGATCCAGCTCCACACCGCGCAACGCAATTAATGTGAG |
| LT87 | CCGGCGTAGAGGATCCGGTGCTGAGTAGTTTCCCATGGATCACTGTCCAGAGACAACAACCCAGCACCTCATTGGGAAGGGCGATCGG |
| LT92 | CCCGTCCTGTGGATCCCGAAAATGTATGGAGGCACTTGTATCAATATAGGATGTATACCTTCGAAGACACTTGCGCAACGCAATTAATGTGAG |
| LT93 | CCGGCGTAGAGGATCCTTGTATGGAAGTTTGTACACTTCGTATTAATGAACTGTTCGTAGTTCCGCACAAAATCATTGGGAAGGGCGATC GG |
| LT88 | CCCGTCCTGTGGATCCGCGCCCAAGTTGCCCATGACCATGCCGAAGCAGTGGTAGAAGGGCACCGGCAGACACGCGCAACGCAATTAATGTGAG |
| LT89 | CCGGCGTAGAGGATCCACCCGCAGAGTGTACCCACAAGCAGTACCACTGCTTCAAGTGGTATCGCTTTGGGGAACATGTCATTGGGAAGGGCGATCGG |
| LT101 | CCCGTCCTGTGGATCTTGTAAAGGAGACTGATAATGGCATGTACAACTATACTCGTCGGTAAAAAGGCAGCGCAACGCAATTAATGTGAG |
| LT102 | CCGGCGTAGAGGATCTTTTAGGCACTACAACTAGTATAGTTGTACATGAAAAACGCTGTATTTTTTTATCATTGGGAAGGGCGATCGG |
| LT94 | CCCGTCCTGTGGATCCCCCGAAAAATTTCGCGTGGATGAGCAATACTTTGATTCAGTGAACCTTTGAAAATCGTGCGCAACGCAATTAATGTGAG |
| LT103 | CCGGCGTAGAGGATCCTATTTTAGGTATATGATTTTGTTTATTAGTGTATATAACACTATGTACCTAAAATTTATTCATTGGGAAGGGCGATCGG |

**Xu et al Additional file**

**Table S3 Assaying integrase activity in human HT1080 cells by deletion activity following transient transfection of integrase expression plasmid**

**Table S3; Additional file.**  The results of two transient transfection experiments in which the integrase shown in the first column was introduced into each of two cell lines containing a single copy of the deletion assay reporter plasmid located intact at a single site in the genome of HT1080 cells.

|  | **Experiment 1** | | | **Experiment 2** | |
| --- | --- | --- | --- | --- | --- |
| **Integrase exprsssion construct** | **Gancyclovir resistant clones** | ***attR* detectable in pooled GANCr cells** | ***attR* detectable in GANCr clones** | **Gancyclovir resistant clones** | ***attR* detectable in pooled GANCr cells** |
| vector | 27 | -ve | Not applicable | 13 | -ve |
| Bxb | 43 | +ve | 1(from8) | 13 | +ve |
| φC31 | 29 | +ve | 2(from8) | 16 | +ve |
| R4 | 28 | +ve | 4(from8) | 14 | +ve |
| φBT | 58 | +ve | 2(from8) | 13 | +ve |
| Wβ | 109 | +ve | 4(from8) | 12 | +ve |
| SPBC | 123 | +ve | 6(from8) | 11 | +ve |
| TP901-1 | 32 | -ve | Not applicable | 11 | +ve |
| TG1 | 31 | -ve | Not applicable | 17 | -ve |
| FC1 | 38 | -ve | Not applicable | 6 | -ve |
| φ370 | 31 | -ve | Not applicable | 16 | -ve |
| K38 | 39 | -ve | Not applicable | 14 | -ve |
| RV | 47 | -ve | Not applicable | 15 | -ve |
| A118 | 52 | -ve | Not applicable | 12 | -ve |
| BL3 | 53 | -ve | Not applicable | 14 | -ve |
| MR11 | 45 | -ve | Not applicable | 13 | -ve |

**Xu et al Additional file**

**Table S4 Assaying integrase activity in human HT1080 cells by deletion activity following genomic integration of integrase expression plasmid**

|  | **Experiment 1** | | | | | | **Experiment 2** | | | |
| --- | --- | --- | --- | --- | --- | --- | --- | --- | --- | --- |
| **Integrase** | **Stably tranfected clones** | **Ganc resistant clones** | **Yield of GANCr clones** | ***attR* detectable in GANCr clones** | ***attR* detectable in HygR clones two weeks further growth in absence of Hyg** | ***attB* and *attP* sites of 2 HygR clones** | **Stably tranfected clones** | **Ganc resistant clones** | **Yield of GANCr clones** | ***attR* detectable in GANCr clones** |
| vector | 1272 | 16 (from636) | 0.02516 | Not applicable | not done |  | 1078 | 11 (from 539) | 0.024 | Not applicable |
| BxB | 157 | 67 (from 83) | 0.807 | 7 (from 10) | not done |  | 129 | 58 (from 65) | 0.89 | 9(from 10) |
| φC31 | 153 | 61 (from 82) | 0.743 | 9 (from 10) | not done |  | 146 | 68 (from74) | 0.92 | 8 (from 10) |
| R4 | 70 | 32 (from 43) | 0.744 | 6 (from 10) | not done |  | 58 | 25 (from 31) | 0.81 | 6 (from 10) |
| φBT | 170 | 47 (from 82) | 0.573 | 8 (from 10) | not done |  | 137 | 54 (from 61) | 0.885 | 8 (from 10) |
| Wβ | 138 | 34 (from 71) | 0.48 | 6 (from 10) | not done |  | 63 | 19 (from 31) | 0.612 | 5(from 10) |
| SPBC | 56 | 22 (from 29) | 0.758 | 8 (from 10) | not done |  | 61 | 26 (from 33) | 0.787 | 5 (from 10) |
| TG1 | 0 | Not applicable | 0 | Not applicable | not done |  | 0 | Not applicable | 0 | Not applicable |
| TP901 | 20 | 3 (from 12) | 0.25 | 1 (from 6*) | 6 (from10*), | no change | 29 | 3 (from 15) | 0.2 | see experiment 1 |
| FC1 | 89 | 37 (from 43) | 0.86 | 0 (from10) | 0 (from10) | no change | 69 | 25 (from 38) | 0.66 | 0 (from10) |
| f370 | 1 | 0 (from 1) | 0 | Not applicable | not done |  | 0 | Not applicable | 0 | Not applicable |
| K38 | 95 | 39 (from 54) | 0.722 | 0 (from10) | 0 (from10) | no change | 115 | 15 (from 60) | 0.25 | 0 (from10) |
| RV | 48 | 4 (from 25),slow | 0.16 | Not done | 0 (from10) | no change | 55 | 12 (from 27) | 0.44 | 0 (from10) |
| A118 | 98 | 29 (from 52), slow | 0.557 | 0 (from10) | 0 (from10) | no change | 144 | 15 (from 78) | 0.192 | 0 (from 10) |
| BL3 | 65 | 15 (from 39), slow | 0.384 | 0 (from10) | 0 (from10) | no change | 89 | 13 (from 48) | 0.27 | 0 (from10) |
| MR11 | 87 | 20 (from 49) | 0.41 | 0 (from10) | 0 (from10) | no change | 84 | 18 (from 39) | 0.46 | 0 (from10) |

**Table S4; Additional file**; this table itemises the results of two experiments in which the indicated integrase expression construct was stably transfected into each of two cell lines containing a single integrated copy of the deletion reporter plasmid; one half of the transfected cells were plated in the presence of gancyclovir to select for those cells that have lost the counter selectable HyTK gene and the remainder in the presence of hygromycin to select for clones in which recombination failed. *Clones from both experiments 1 and 2 were analysed in order to make up the numbers.

**Xu et al Additional file**

**Table S5 Assaying integrase activity in mouse ES cells by deletion activity following transient transfection of integrase expression plasmid**

|  | **Experiment 1** | | | **Experiment 2** | | | |
| --- | --- | --- | --- | --- | --- | --- | --- |
| **Integrase expression construct** | **GancR clones** | **Neo^R^ colonies (transfection control)** | **Normalized no. of Ganc^R^ clones** | **Ganc^R^ clones** | **Neo^R^ colonies (transfection control)** | **Normalized no. of Ganc^R^ clones** | **PCR(attR): single colony** |
| vector | 44 | 16 | 2.8 | 9 | 16 | 0.6 | not done |
| BxB | 1349 | 24 | 56.2 | 1827 | 26 | 70.3 | 5 (from 8) |
| φC31 | 1879 | 42 | 44.7 | 1757 | 39 | 45.1 | 4 (from 8) |
| R4 | 1235 | 36 | 34.3 | 922 | 23 | 40.1 | 3 (from 12) |
| φBT | 148 | 21 | 7.0 | 183 | 39 | 4.7 | 6 (from 11) |
| Wβ | 3323 | 23 | 144.5 | 2723 | 18 | 151.3 | 6 (from 8) |
| SPBC | 1191 | 29 | 41.1 | 1305 | 32 | 40.8 | 6 (from 11) |
| TG1 | 1105 | 11 | 100.5 | 1053 | 9 | 117.0 | 1 (from 12) |
| TP901 | 818 | 16 | 51.1 | 1061 | 23 | 46.1 | 0 (from 12) |
| FC1 | 78 | 35 | 2.2 | 52 | 28 | 1.9 | 0 (from 4) |
| φ370 | 0 | 7 | 0.0 | 9 | 6 | 1.5 | 0 (from 4) |
| K38 | 244 | 22 | 11.1 | 104 | 17 | 6.1 | 0 (from 4) |
| RV | 35 | 19 | 1.8 | 26 | 11 | 2.4 | 0 (from 4) |
| A118 | 44 | 23 | 1.9 | 122 | 34 | 3.6 | 0 (from 4) |
| BL3 | 26 | 34 | 0.8 | 26 | 21 | 1.2 | 0 (from 4) |
| MR11 | 35 | 26 | 1.3 | 104 | 36 | 2.9 | 0 (from 4) |

**Table S5; Additional file.**  The results of two transient transfection experiments in which a plasmid directing expression of the integrase shown in the first column was introduced into each of a single ES cell line containing a single copy of the deletion assay reporter plasmid targeted to the ROSA26 locus and deletion activity measured by the number of gancyclovir resistant clones. In order to control for the effects of integrase expression upon transfection efficiency the cells were co-transfected with a linearized PGK neo plasmid and the numbers of colonies generated following G418 selection (columns 3 and 6) was used to normalize the yields of gancyclovir resistant clones (columns 2 and 5).

**Xu et al Additional file**

**Table S6 Assaying site-specific integration activity of seven integrases able in human HT1080 cells.**

|  |  | **Experiment 1** | | | | **Experiment 2** | | | | **Experiment 3** | | | |
| --- | --- | --- | --- | --- | --- | --- | --- | --- | --- | --- | --- | --- | --- |
| **Integrase** | **Trasfected plasmid** | **No of G418 resistant colonies** | **No. of Neor/ GANCr colonies** | **No. of Neor/ GANCr colonies/ no colonies with CCAGneo** | **attR+L in clones** | **No of G418 resistant colonies** | **No. of Neor/ GANCr colonies** | **No. of Neor/ GANCr colonies/ no colonies with CCAGneo** | **attR+L in clones** | **No of G418 resistant colonies** | **No. of Neor/ GANCr colonies** | **No. of Neor/ GANCr colonies/ no colonies with CCAGneo** | **attR+L in clones** |
| Empty vector | CCAGNEO | 18 | 0 |  |  | 7 | 0 |  |  | 25 | 0 |  |  |
| Empty vector | attBCCAGneoattB | 26 | 1 | 0.05 |  | 15 | 0 | 0 |  | 48 | 2 | 0.1 |  |
| BxB | CCAGNEO | 53 | 0 |  |  | 58 | 1 |  |  | 35 | 1 |  |  |
| BxB | attBCCAGneoattB | 88 | 75 | 1.42 | 8 (from8) | 88 | 81 | 1.4 | 7 (from8) | 82 | 79 | 2.26 | 10 (of 10) |
| φC31 | CCAGNEO | 39 | 2 |  |  | 29 | 1 |  |  | 29 | 1 |  |  |
| φC31 | attBCCAGneoattB | 51 | 36 | 0.92 | 8 (from8) | 41 | 26 | 0.9 | 6 (from8) | 44 | 28 | 0.96 | 8 (of 10) |
| R4 | CCAGNEO | 41 | 1 |  |  | 41 | 0 |  |  | 48 | 0 |  |  |
| R4 | attBCCAGneoattB | 59 | 23 | 0.45 | 2 (from8) | 59 | 25 | 0.61 | 3 (from23) | 60 | 34 | 0.708 | 10 (of 10) |
| φBT | CCAGNEO | 8 | 0 |  |  | 19 | 0 |  |  | 21 | 0 |  |  |
| φfBT | attBCCAGneoattB | 13 | 2 | 0.25 | 1 (from8) | 26 | 2 | 0.11 | 2 (from23) | 27 | 7 | 0.33 | 2 (of 7) |
| Wβ | CCAGNEO | 71 | 7 |  |  | 66 | 3 |  |  | 53 | 1 |  |  |
| Wβ | attBCCAGneoattB | 75 | 49 | 0.69 | 0 (from8) | 75 | 65 | 1 | 0 (from23) | 80 | 71 | 1.33 | 0 |
| SPBC | CCAGNEO | 9 | 0 |  |  | 19 | 4 |  |  | 5 | 0 |  |  |
| SPBC | attBCCAGneoattB | 10 | 7 | 0.77 | 0 (from8) | 38 | 29 | 1.52 | 0 (from23) | 13 | 5 | 1 |  |
| TP901 | CCAGNEO | 38 | 7 |  |  | 34 | 3 |  |  | 49 | 2 |  |  |
| TP901 | attBCCAGneoattB | 51 | 21 | 0.55 | 0 (from8) | 51 | 21 | 0.61 | 0 (from23) | 84 | 61 | 1.24 | 0 (from 10) |

**Table S6; Additional file.** The results of three assays of site-specific integration activity of seven integrases able in human HT1080 cells. A single intact *attP* array CCAG HyTK *attP*  array integration reporter was introduced into the genome of HT1080 cells by electroporation and two independent clones were then stably transfected with the indicated integrase expression construct. Two independent integrase expressing clones were then used in each of the three integration assays in which the integration donor plasmid *attB* array CCAG Neo *attB* array was transiently transfected into cells of these two clones and scored for G418 and gancyclovir resistance. Experiments 1 and 2 used the same clone.

**Xu et al Additional file**

**Table S7 Primers used to analyse re-arrangements associated with cassette exchange integrations: illustrated in figure 6 of main text.**

| **Reaction** | **Template** | **forward primer** | **Sequence** | **reverse primer** | **Sequence** |
| --- | --- | --- | --- | --- | --- |
| **1** | attParray CCAG HyTK attParray | ZXY360 | CAGGAAACAGCTATGACCATG | ZXY394 | GTCGTGGTTTGTCTGGTCAACC |
| **2** | attParray CCAG HyTK attParray | ZXY398 | AATATCACAGTAACTAATAACCA | WRAB129 | AACTAATGACCCCGTAATTGATTAC |
| **3** | attParray CCAG HyTK attParray | ZXY072 | CCTCTGCTAACCATGTTCATGC | WRAB061 | GGGAGATGGGGGAGGCTAACTG |
| **4** | attParray CCAG HyTK attParray | ZXY069 | GCGACCTGGCGCGCACGTTTGCC | ZXY394 | CCAACTGGGTAAGAGCAAAGACT |
| **5** | attParray CCAG HyTK attParray | ZXY398 | GATACTTACTACTCCAACTCGCT | ZXY359 | GGATAACAATTTCACACAGGAG |
| **A** | attBarray CCAGneo attBarray | ZXY360 | CAGGAAACAGCTATGACCATG | ZXY335 | GCGGAGCTGATCGCCCGGAT |
| **B** | attBarray CCAGneo attBarray | ZXY360 | CAGGAAACAGCTATGACCATG | ZXY404 | GTTCCCTCTTTGCGTCCTTCATA |
| **C** | attBarray CCAGneo attBarray | ZXY359 | GGATAACAATTTCACACAGGAG | ZXY404 | GTTCCCTCTTTGCGTCCTTCATA |
| **D** | attBarray CCAGneo attBarray | ZXY359 | GGATAACAATTTCACACAGGAG | ZXY403 | TCTCTCGTGGTGGTGGAAGGTGTT |
| **1049 BNB/4** | re-arranged array | ZXY360 | CAGGAAACAGCTATGACCATG | ZXY359 | GGATAACAATTTCACACAGGAG |
| **1051 BNB/4,9** | re-arranged array | ZXY383 | GGTTGTTGTCTCTGGACAGTGAT | ZXY399 | TCGTAGTTCCGCACAAAAGAAGT |

**
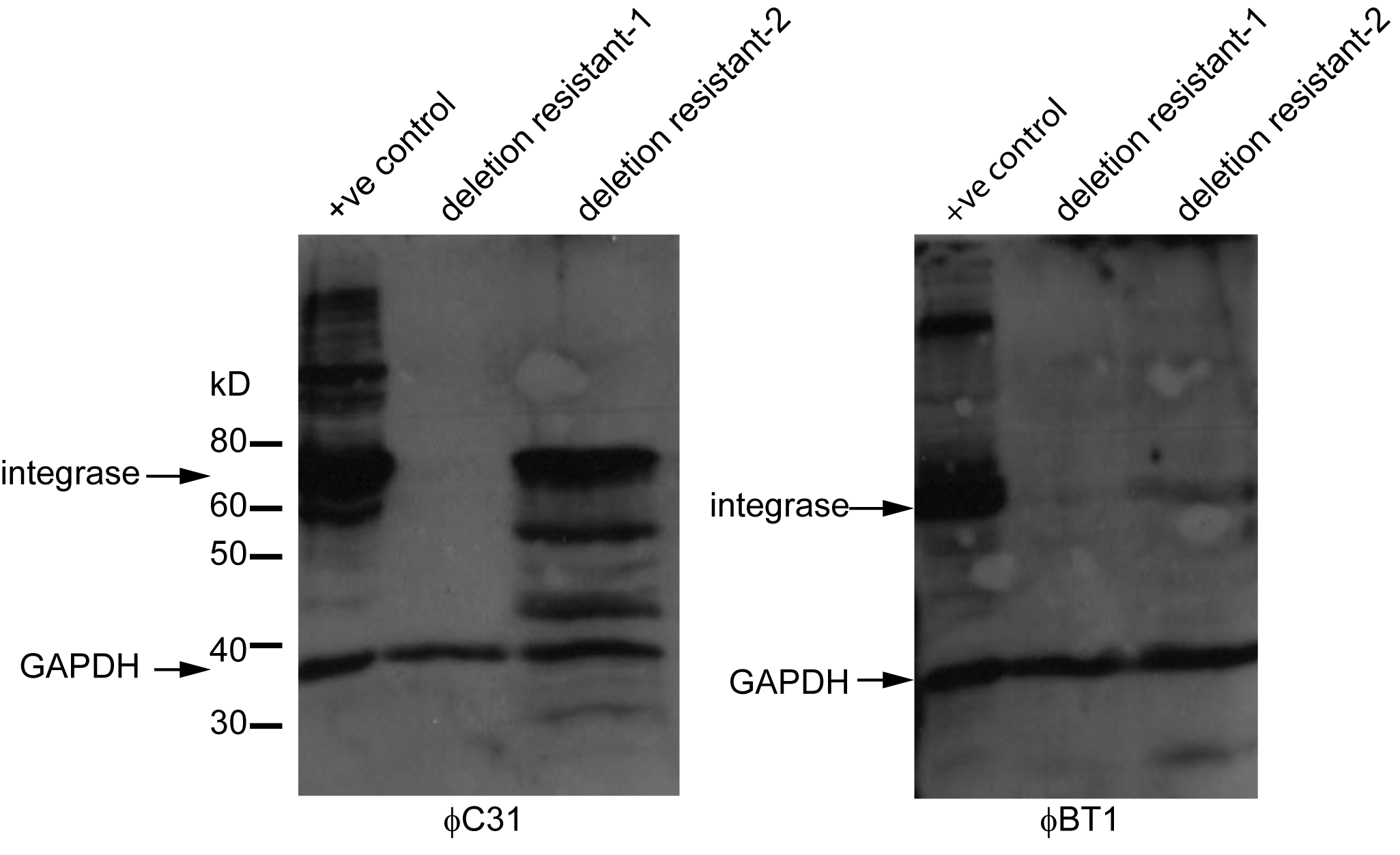
**

**Figure S1 Additional file. (A)** Targeting the ROSA26 locus in mouse ES cells with a deletion reporter construct. **(B)** the targeted locus before deletion **(C)** the targeted locus after deletion. Sequences of the Del-Rosa primers are in the main text.

C

B

A

**Figure S2 Additional file:** western blot analysis of φC31 and φBT1 integrase expression in HT1080 clones that contain a single integrated copy of the *attP* array CCAG HyTk *attB* array reporter and have been stably transfected with an integrase expression plasmid but fail to delete the HyTk gene. The positive controls were integrase expressing *attP* array CCAG HyTk *attP* array clones that stably express functional integrase as determined by their ability to integrate a *attB* array CCAG neo *attB* reporter. Indicated are the positions of the respective integrase and the glyceraldehyde-3-phosphate loading control.
